# Burden of Vitiligo in Canada: Retrospective Analysis of a Canadian Public Claims Database

**DOI:** 10.1177/12034754241304683

**Published:** 2025-01-22

**Authors:** Julien Ringuet, Grace K. Wong, Véronique Baribeau, Sunil Kalia, Josée Brisebois, Jean Lachaine

**Affiliations:** 1 Centre de Recherche Dermatologique du Québec Métropolitain, Québec City, QC, Canada; 2 Incyte Biosciences Canada Corporation, Pointe-Claire, QC, Canada; 3 PeriPharm Inc, Montréal, QC, Canada; 4 Department of Dermatology and Skin Science and Photomedicine Institute, University of British Columbia, Vancouver, BC, Canada; 5 Faculty of Pharmacy, Université de Montréal, Montréal, QC, Canada

**Keywords:** HCRU, treatment patterns, vitiligo

## Abstract

**Background:**

Vitiligo is an autoimmune disease resulting in skin depigmentation. Treatment options are limited.

**Objectives:**

To examine disease burden and healthcare resource utilization (HCRU) among patients with vitiligo in Québec, Canada.

**Methods:**

In this retrospective study, data were obtained from the Régie de l’Assurance Maladie du Québec (RAMQ) databases for 125,000 random individuals from January 2010 to December 2019. The *International Classification of Diseases, Ninth Revision* (*ICD-9*) diagnostic code [709.x (other skin disorders)] with vitiligo-related treatment was used to identify patients with vitiligo. Patient characteristics and treatments, including treatment type, episodes (treatments used without discontinuation), and sequences (treatment episodes ≥30 days), were assessed. Annualized HCRU and costs (2021 adjusted) included all-cause hospitalization, emergency department visits, outpatient visits, and medications among patients with vitiligo (n = 113) and age- and sex-matched non-vitiligo controls (n = 339).

**Results:**

Of patients with vitiligo (mean age, 50.0 years; 68.1% female) identified using *ICD-9* code 709.x with vitiligo-related treatment, 36.3% received ≥4 treatment episodes. Treatment patterns were heterogeneous, with 43 different sequences reported. Annualized mean outpatient visits (16.1 vs 5.5) and all-cause outpatient service costs per patient were significantly higher in the vitiligo versus the control group (CAN$1037 vs CAN$523; *P* < .01). Total all-cause services costs were higher for patients with vitiligo in the year after versus before diagnosis (CAN$3679 vs CAN$2085; *P* = .04).

**Conclusions:**

Vitiligo is associated with significant burden and HCRU among patients in Québec, Canada, who were identified by *ICD-9* code 709.x plus vitiligo-related treatment. Measurement of true vitiligo burden remains challenging.

## Introduction

Vitiligo is a chronic autoimmune disease that results in melanocyte destruction and skin depigmentation.^1^ Onset commonly occurs before age 30 years, although it may occur at any age.^2^ Patients with vitiligo have a higher prevalence of other autoimmune diseases^3,4^ and mental health comorbidities, such as depression and anxiety^5^; patients with vitiligo have increased hospitalizations for mental health disorders.^6^ The majority of patients with vitiligo report a moderate to severe effect on the overall quality of life (QoL), which is exacerbated by certain disease characteristics, such as greater disease extent [ie, high body surface area (BSA) involvement] and involvement of special body areas (eg, face, genitals, and hands).^7,8^

Conventional treatment options for vitiligo include topical corticosteroids (TCS), topical calcineurin inhibitors, phototherapy, and systemic immunosuppressants.^9,10^ Among newer treatment options, ruxolitinib cream is the first treatment approved for repigmentation of vitiligo^9^ in the United States,^11^ Canada,^12^ European Union,^13^ and United Kingdom.^14^ Combination therapies are commonly prescribed for vitiligo (eg, phototherapy, topicals, and systemic treatments may be combined in various settings).^9^

The prevalence of vitiligo in children and adults worldwide is estimated at 0.5% to 2.0%.^8,15^ Vitiligo may be underreported or undertreated because its burden may be erroneously deemed asymptomatic or cosmetic by providers or payers rather than recognized as an autoimmune disease that can be managed with appropriate treatment.^16,17^ The global VALIANT study showed that 45% of patients were previously misdiagnosed, and the average time from first lesions to formal vitiligo diagnosis was 2.4 years.^18^ To the best of our knowledge, there are no documented nationwide prevalence rates for vitiligo in Canada. There are also limited data regarding epidemiology, treatment patterns, and healthcare resource utilization (HCRU) among patients with vitiligo in Canada. As such, this retrospective study aimed to evaluate disease burden and HCRU for patients with vitiligo in Québec, Canada, using claims data from the Régie de l’Assurance Maladie du Québec (RAMQ).

## Materials and Methods

### Data Source

Data for this study were obtained from the RAMQ, the government agency responsible for public health coverage in Québec. Similar to other Canadian provinces, Québec has a universal healthcare program for physician services and hospitalizations that covers the entire population and a public drug plan for those without private insurance (ie, aged ≥65 years, beneficiaries of social support, or without access to a private plan) that covers a large portion of the population (3.7 million people in 2020-2021).^19^ The RAMQ has a medical services database that contains physicians’ claims for in-hospital and out-of-hospital services and a pharmaceutical services database that contains pharmacists’ claims for dispensed medication that was reimbursed by the public drug plan (but not medication received in the hospital). RAMQ databases also include an encrypted patient identifier (anonymously linking patient information), demographic information of each insured person (eg, age, sex, and geographic location), and physician information (eg, specialty). Because this is an analysis of claims using anonymized data from RAMQ, ethics and institutional review board evaluation and approval was not required for this observational, retrospective study.

### Patient Selection

A random sample of 125,000 patients in the RAMQ databases between January 2010 and December 2019 was used to identify patients with vitiligo and a non-vitiligo matched control group who received ≥1 medical service between January 1, 2018 and December 31, 2019. Cases of vitiligo were identified as either ≥1 diagnosis of vitiligo [*International Classification of Diseases, Tenth Revision* (*ICD-10*) code: L80.x] or as ≥1 diagnosis of other skin disorders [*International Classification of Diseases, Ninth Revision* (*ICD-9*) code: 709.x] combined with ≥1 treatment related to vitiligo (tacrolimus, pimecrolimus, or phototherapy, according to a validated algorithm^20^) between January 2010 and September 2019. The *ICD-9* code 709.x for other skin disorders includes dyschromia (eg, vitiligo), vascular disorders, scar conditions, degenerative disorders, foreign body granulomas, and other specified and unspecified disorders of the skin and subcutaneous tissue, but the case definition used differentiates vitiligo from these conditions. Also, other skin diseases that may be treated with the targeted treatments in the algorithm (eg, psoriasis, atopic dermatitis, and lichenoid dermatitis) have their own *ICD-9* codes and are therefore not included. The index date for the patient group was defined as the first diagnosis date of the first other skin disorder (*ICD-9* code) or vitiligo (*ICD-10* code) recorded in the database.

Eligible individuals in the control group had no diagnosis of other skin disorders (*ICD-9* code 709.x) or vitiligo (*ICD-10* code L80.x) between January 2010 and September 2019. The index date for the control group was defined as the first pharmaceutical service recorded during this period.

All individuals included in the study were required to be covered by the RAMQ Drug Plan Insurance for ≥3 months after the index date to allow for follow-up. The follow-up period ended either at the end of coverage or by the end of the study period, whichever came first.

### Study Design and Data Analysis

This was a retrospective, observational, cohort study using data from 2010 to 2019 collected from the RAMQ databases. Extracted data include demographics and comorbidities as well as all medical services and drugs reimbursed during the study period. The exact age of individuals was not provided by RAMQ; instead, each patient was assigned an age group based on age at the index date (ie, <1, 1-4, 5-9, every 5 years up to age 84, and ≥85 years); ages were estimated as the middle of the age group. Comorbidities were described for all patients in the vitiligo group and non-vitiligo matched control group in the year after their respective index dates based on ≥1 *ICD-9/10* code. Comorbidity severity was estimated by the Charlson comorbidity index^21^; higher score indicates greater severity. Incidence and prevalence of vitiligo were estimated from the selected population and extrapolated to the Québec population. Prevalence of vitiligo was assessed in December 2019 and was based on a diagnosis of vitiligo^20^ within the previous 5 years and required ≥1 medical or pharmaceutical service from December 2018 to December 2019 (indicating patients were alive). Incidence was estimated yearly; patients were required to have a new diagnosis of vitiligo (according to the algorithm) in a given year with no diagnosis in the previous year.

Treatment utilization related to vitiligo was described in terms of type of treatment, sequence of treatment, concomitance, and seasonality. One episode of treatment was defined as a period of treatment without discontinuation; a patient could have >1 episode for the same type of treatment if it was restarted after discontinuation (gap of dispensation for ≥90 days). A dispensation gap of ≥180 days was used in a sensitivity analysis. Treatment episodes ≥30 days were considered part of a sequence. Adherence to treatment was estimated by the medication possession ratio (MPR), which was calculated as the total number of days’ supply of medication dispensed (estimated by the dispensing pharmacist) divided by the length of follow-up in patients who had not discontinued treatment and who were covered by the drug plan at each specific follow-up point. MPR was dichotomized, with a threshold of ≥80% indicating adherent patients. Persistence to treatment was defined as the number of days of use during the first treatment episode for each treatment.

HCRU data were reported on an annualized basis as well as the year before and after the index date and before and after treatment for all-cause resources and mental health resources (*ICD-9* codes 290-319 or *ICD-10* codes F00-F99) in the vitiligo and non-vitiligo control groups. HCRU of vitiligo-related resources (*ICD-9* code of other skin disorders or *ICD-10* code of vitiligo) was reported only for patients in the vitiligo group. HCRU comprised the number and cost of hospital services [inpatient visits, emergency department (ED) visits, outpatient visits, and other visits], total medication cost (excluding in-hospital medications), total medical service cost, and total healthcare cost. Reported location of medical activity and time between claims were used to estimate the number and length of inpatient admissions, ED visits, and outpatient visits (general practitioners and specialists) from RAMQ medical claims. Only 1 inpatient visit was counted if the time between 2 inpatient claims was ≤7 days. One ED visit was counted if claims had the same date. Costs for visits were estimated using the average daily cost of an inpatient visit in 2016 (CAN$1369), an ED visit in 2012 (CAN$161.38), or an outpatient visit in 2012 (CAN$29.82) and adjusted to 2021 values using the annual consumer price index of Québec.^22^

### Statistical Analysis

IBM SPSS Statistics software [version 25 (IBM Corp., Armonk, NY, USA)] was used for all data analyses. Patients with vitiligo and the matched control group [up to 3 control patients for each vitiligo case, matched for age group (<20, 20-34, 35-49, 50-64, ≥65 years) and sex] were compared for patient characteristics and HCRU using independent *t* tests for continuous variables and chi-square tests for categorical variables. HCRU before and after index date and before and after treatment were compared with paired *t* tests.

## Results

### Patients

The vitiligo group included 113 patients, all of whom were identified by an *ICD-9* code of other skin disease and ≥1 treatment related to vitiligo (Figure S1). For the age-and-sex-matched non-vitiligo control group (received ≥1 medical service in 2018-2019), 339 patients were selected in a 1:3 ratio (Figure S1). Some geographical differences were reported between the vitiligo and the non-vitiligo–matched control group (Table S1). The mean (SD) age among patients with vitiligo was 50.0 (24.9) years, and 68.1% were female.

### Vitiligo Incidence and Prevalence

**Figure 1.**
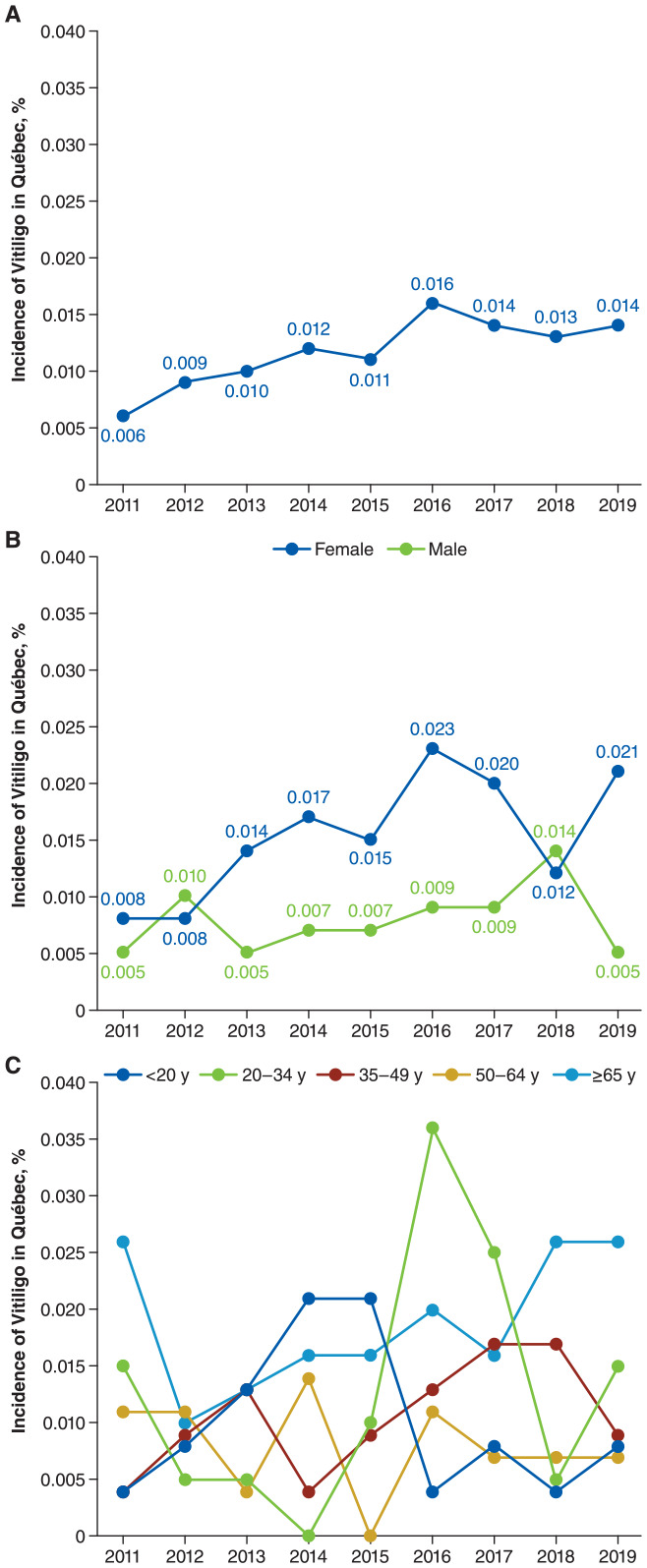
Incidence of vitiligo identified by algorithm^a^ in Québec(A) overall and by (B) sex and (C) age group. ^a^Cases of vitiligo were identified as ≥1 diagnosis of other skin disorders (International Classification of Diseases, Ninth Revision code: 709.x) combined with ≥1 treatment related to vitiligo.

New cases of vitiligo presenting to physicians identified via the algorithm (*ICD-9* code 709.x plus vitiligo-related treatment) in Québec doubled between 2011 and 2019 (0.006% vs 0.014%; Figure 1A) and was generally higher among female patients and patients ≥65 years old (Figure 1B and C). Prevalence of vitiligo determined using the algorithm was estimated to be 0.065% in 2019 and was higher among female versus male patients (0.086% vs 0.041%, respectively) and patients ≥65 years old [0.108% vs <20 years (0.042%), 20-34 years (0.071%), 35-49 years (0.065%), 50-64 years (0.032%)].

### Clinical Characteristics

Comorbidities in the year following the index date were generally similar between the vitiligo and control groups (Table 1). The vitiligo group had a significantly higher proportion of skin disorders versus the control group (11.5% vs 2.1%, respectively; *P* < .01), particularly psoriasis (6.2% vs 0.3%; *P* < .01). The control group had a significantly greater proportion of depression than the vitiligo group (4.1% vs 0%, respectively; *P* = .03). The Charlson comorbidity index was similar between patients with vitiligo and the control group in the year following their respective index dates [mean (SD), 0.37 (0.93) vs 0.36 (1.10), respectively; *P* = .94].

**Table 1.** Comorbidities in the Year Following Index Date.^a^

Comorbidity,^b^ n (%)	Vitiligo (n = 113)	Control (n = 339)	*P* value
Autoimmune diseases	5 (4.4)	5 (1.5)	.13
Multiple sclerosis	2 (1.8)	2 (0.6)	.26
Inflammatory bowel disease	1 (0.9)	2 (0.6)	1.00
Rheumatoid arthritis	1 (0.9)	1 (0.3)	.44
Myasthenia gravis	1 (0.9)	0	.25
Systemic lupus erythematosus	1 (0.9)	0	.25
Idiopathic thrombocytopenic purpura	0	0	—
Mental health disorders	16 (14.2)	51 (15)	.82
Anxiety	10 (8.8)	24 (7.1)	.54
Depression	0	14 (4.1)	.03*
Sleep disturbances	2 (1.8)	7 (2.1)	1.00
Adjustment disorders	2 (1.8)	6 (1.8)	1.00
Schizophrenia	2 (1.8)	3 (0.9)	.60
Personality disorder	0	4 (1.2)	.58
Childhood psychiatric disorders	1 (0.9)	2 (0.6)	1.00
Attention deficit/hyperactivity disorder	1 (0.9)	1 (0.3)	.44
Eating disorders	1 (0.9)	1 (0.3)	.44
Parkinsonism	0	2 (0.6)	1.00
Substance use disorder	1 (0.9)	1 (0.3)	.44
Conduct disorder	0	0	—
Obsessive-compulsive disorder	0	0	—
Metabolic comorbidities	25 (22.1)	83 (24.5)	.61
Hypertension	13 (11.5)	50 (14.7)	.44
Diabetes	9 (8.0)	23 (6.8)	.67
Dyslipidemia	4 (3.5)	22 (6.5)	.35
Obesity	2 (1.8)	2 (0.6)	.26
Other diseases	1 (0.9)	12 (3.5)	.20
Thyroid disorders	1 (0.9)	8 (2.4)	.33
Breast cancer	0	3 (0.9)	.58
Lymphoma	0	1 (0.3)	1.00
Thyroid cancer	0	0	—
Skin disorders	13 (11.5)	7 (2.1)	<.01**
Psoriasis	7 (6.2)	1 (0.3)	<.01**
Acne	2 (1.8)	2 (0.6)	.26
Urticaria	1 (0.9)	2 (0.6)	1.00
Atopic dermatitis	2 (1.8)	0	.06
Herpes zoster	1 (0.9)	2 (0.6)	1.00
Charlson comorbidity index, mean (SD)	0.37 (0.93)	0.36 (1.10)	.94

Abbreviations: *ICD-9*, *International Classification of Diseases, Ninth Revision; ICD-10*, *International Classification of Diseases, Tenth Revision*.

a The index date for the patient group was defined as the first diagnosis date of the first other skin disorder (*ICD-9* code) or vitiligo (*ICD-10* code) recorded in the database. The index date for the control group was defined as the first pharmaceutical service recorded during the study period.

b Patients may have >1 comorbidity per category.

* *P* < .05. **P < .01.

### Treatment Characteristics

**Figure 2.**
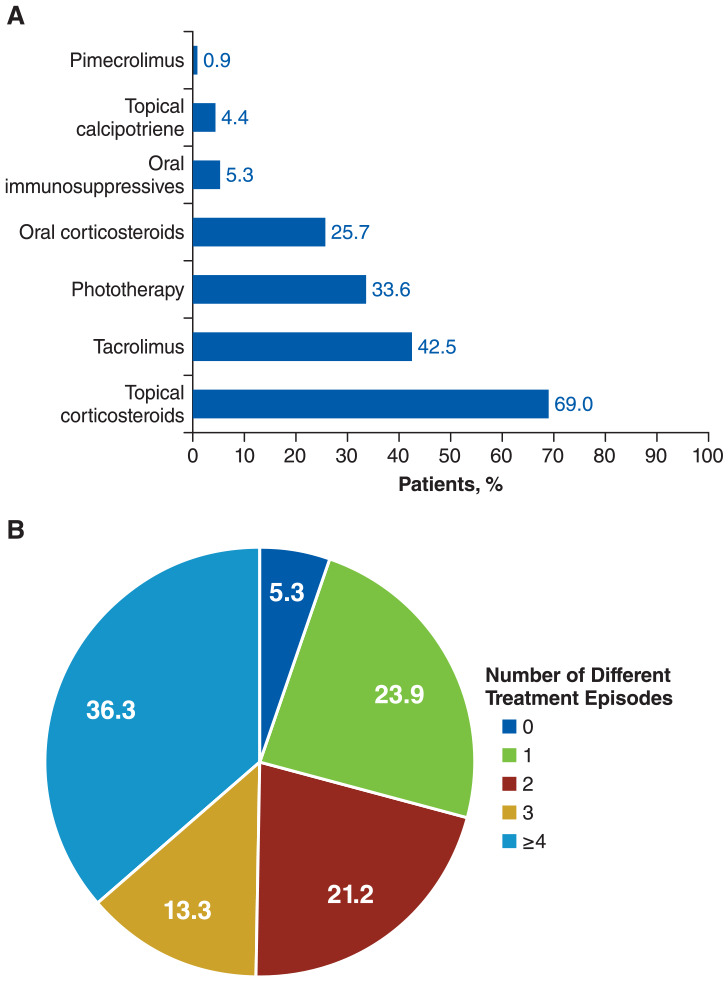
(A) Treatments and (B) number of treatment episodes^a^ for patients with vitiligo (N = 113). ^a^Treatment episode is defined as any treatment used without discontinuation. A patient could restart a treatment after discontinuation and have another treatment episode for the same treatment. Treatment episodes after diagnosis date (index date) are shown.

The most common treatments were TCS (69.0%), followed by tacrolimus (42.5%) and phototherapy (33.6%; Figure 2A). The majority of these treatments were prescribed by dermatologists (TCS: 51.9%; tacrolimus: 83.1%; phototherapy: 100%); prescriptions from general practitioners were more common for dexamethasone (79.2%), prednisone (67.3%), and methotrexate (53.3%), although few patients were prescribed dexamethasone (n = 3) and methotrexate (n = 5). Most patients (94.7%) received ≥1 treatment episode, and many (36.3%) received ≥4 treatment episodes (Figure 2B). TCS was the most common first- (46.7%), second- (58.8%), and third-line (46.4%) treatment. Treatment patterns did not change when analyzed by each month of the year, although some variation was observed for dexamethasone, topical calcipotriene, and cyclosporine, likely owing to the small number of prescriptions (Figure S2). Among patients with vitiligo, 43 different treatment sequences were reported (Table S2). The most common treatment sequences were no sequence (ie, no treatment lasting ≥30 days; 27.4%), phototherapy only (10.6%), and TCS only (8.8%). Among patients undergoing concomitant treatment, the most common treatment combinations were phototherapy plus TCS (31.4%), tacrolimus plus TCS (22.9%), and prednisone plus TCS (14.3%). Treatment adherence and persistence rates were generally low across all treatments with a ≥90-day dispensation gap (Table S3), with similar rates per sensitivity analysis with a ≥180-day dispensation gap (Table S4). The mean MPR for TCS was 57.9%, 65.8%, and 74.7% at 3, 6, and 12 months, respectively; the discontinuation rate was 65.5%, 86.2%, and 93.8%. At 12 months, all treatments had discontinuation rates >90% except methotrexate (50.0%) and topical calcipotriene (80.0%).

### Healthcare Resource Utilization

**Figure 3.**
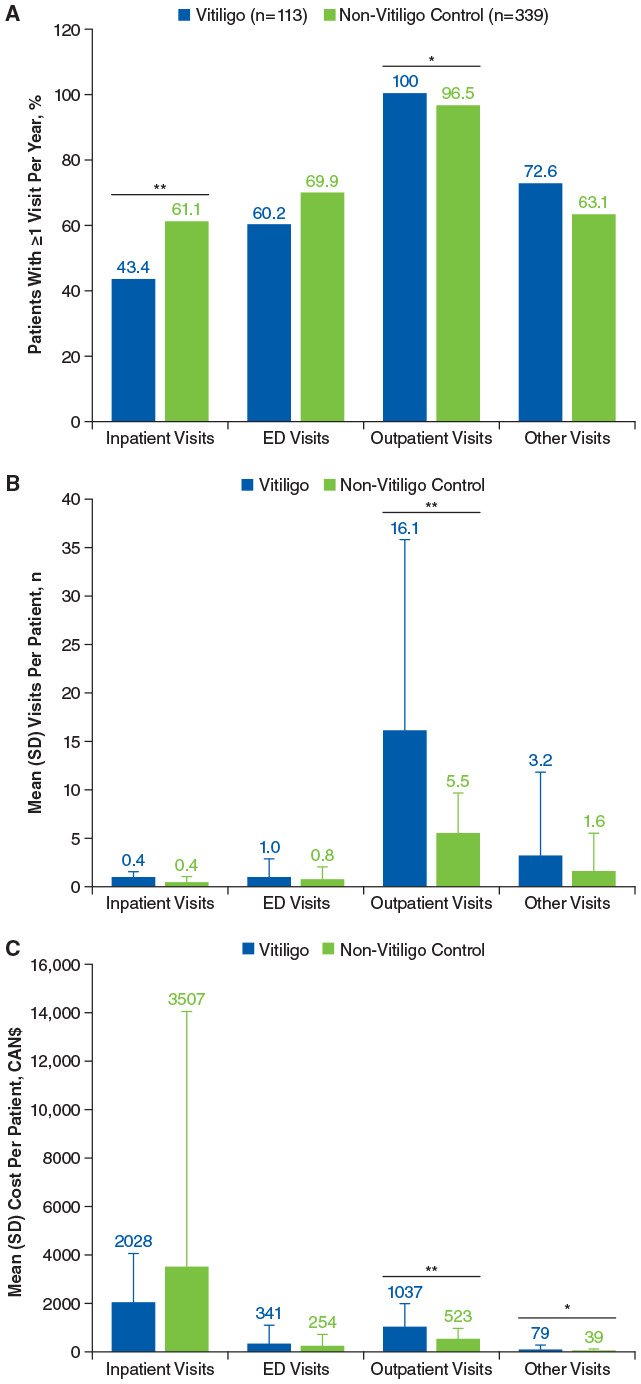
Annualized (A) healthcare resource utilization, (B) mean visits per patient, and (C) mean costs for all-cause resources during the follow-up period in patients with vitiligo and non-vitiligo matched control group. ED, emergency department. *P < .05. **P < .01.

All patients in the vitiligo group had ≥1 outpatient visit per year versus 96.5% of individuals in the matched control group (*P* < .05; Figure 3A). Patients with vitiligo had a significantly higher number of all-cause outpatient visits per patient versus the control group [mean (SD), 16.1 (19.8) vs 5.5 (4.2), respectively; *P* < .01; Figure 3B], as well as outpatient service costs per patient [mean (SD), CAN$1037 (951) vs CAN$523 (458), respectively; *P* < .01; Figure 3C]. Although more patients in the non-vitiligo control group versus vitiligo group had ≥1 inpatient visit and ED visit per year (Figure 3A), the number of visits per patient was similar between the 2 groups (Figure 3B). The cost for other visits was also significantly higher for patients with vitiligo [mean (SD), CAN$79 (188) vs CAN$39 (96); *P* = .03], although the difference for number of visits was not statistically significant. The annual total HCRU was similar between the vitiligo and control groups [mean (SD), CAN$6148 (12,190) vs CAN$5682 (11,669), respectively; *P* = .72]. The mean (SD) total annualized services cost related to vitiligo was CAN$172 (523), and the total annualized medications cost was CAN$90 (189; Table S5). Mental health-related HCRU were generally similar between patients with vitiligo and the control group.

**Figure 4.**
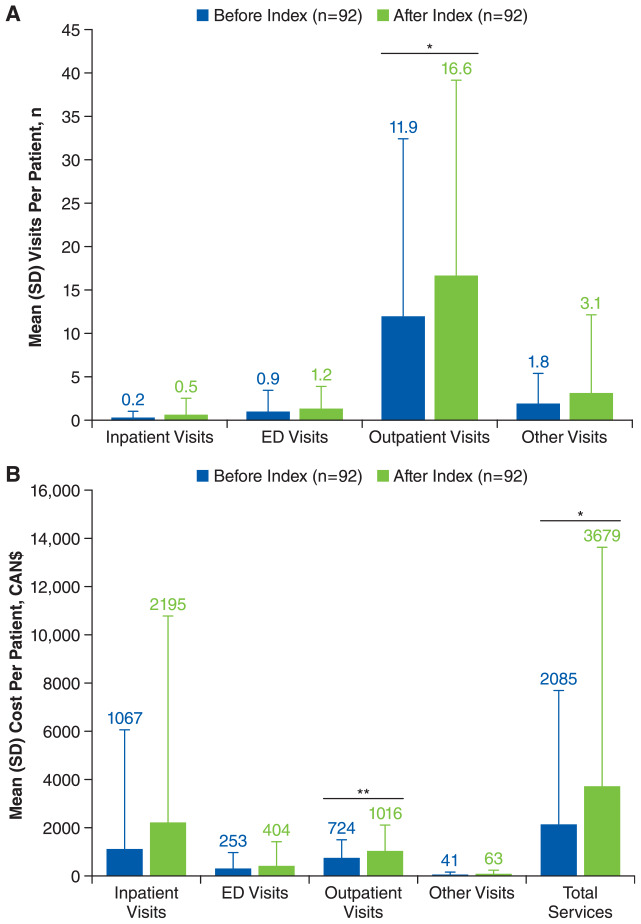
Mean (A) healthcare resource utilization and (B) costs for all-cause resources before and after index date in patients with vitiligo.^a^ ED, emergency department. ^a^Only includes patients with ≥1 year of data before and after the index date.

HCRU was higher after versus before the index date among patients with vitiligo (Figure 4A and B). The number of all-cause outpatient visits per patient was significantly higher in the year after the index date versus the year before [mean (SD), 16.6 (22.5) vs 11.9 (20.5), respectively; *P* = .03], as were outpatient costs per patient [mean (SD) CAN$1016 (1067) vs CAN$724 (752), respectively; *P* < .01]. Total all-cause services costs were also significantly higher for patients with vitiligo in the year after the index date versus the year before [mean (SD), CAN$3679 (9955) vs CAN$2085 (5583), respectively; *P* = .04]. Similarly, outpatient visits per patient were higher after the start of treatment versus before [mean (SD), 25.3 (33.2) vs 7.8 (6.8), respectively; *P* < .01], as were the costs of outpatient visits [mean (SD), CAN$1183 (1260) vs CAN$720 (779), respectively; *P* < .01].

## Discussion

This retrospective, observational, cohort study analyzed the incidence and prevalence of vitiligo identified through the algorithm of *ICD-9* code 709.x plus vitiligo-related treatment in the RAMQ database, as well as clinical characteristics, treatment characteristics, and HCRU of patients treated for vitiligo in Québec over a 10-year period. Data obtained from the RAMQ databases allow the linking of Québec’s universal health program with the public drug plan and thus partially reflects real-world practices.

Using the algorithm, the prevalence of patients receiving treatments related to vitiligo was found to be 0.065%, which does not correlate with the reported prevalence of vitiligo in most countries^8,15^ and is likely an underestimation. Possible reasons for lower reported rates may include barriers to patients accessing healthcare, undertreatment of vitiligo by healthcare providers, lack of effective treatment options for vitiligo, lack of reimbursement for vitiligo treatments, and limited identification of patients with vitiligo by healthcare databases (no patients were identified using the *ICD-10* code L80.x for vitiligo). The limited number of patients with vitiligo identified in this analysis of 125,000 random patients within the RAMQ database may have also contributed to an underestimation of vitiligo prevalence.

Regarding comorbidities, only psoriasis was significantly more prevalent in patients with vitiligo versus the control group. Other studies have reported additional comorbidities that are significantly associated with vitiligo.^3,4,23^ The lack of association of vitiligo with anxiety and depression is particularly surprising given that these comorbidities have been extensively reported in the literature.^5^ Of note, the control group consisted of individuals from the RAMQ database (as opposed to a healthy population) who had received ≥1 medical service between January 2018 and December 2019. Indeed, the RAMQ control cohort had more patients with ≥1 inpatient or ED visit per year versus the identified vitiligo cohort. It is possible that individuals in the control group received medical services for psychologic stress, a frequent cause of consultation that may have skewed the rate of depression within the group. In addition, because it was not possible to identify special site involvement or the extent of the disease (ie, high BSA), the probability of psychiatric comorbidities may have been reduced as those characteristics are usually associated with a higher psychosocial burden.^5^ Finally, as the vitiligo cohort comprised patients who were receiving treatment, psychosocial burden may have been underestimated because this analysis did not assess patients with vitiligo who were not undergoing treatment and may have a higher psychosocial burden. The small size of the vitiligo cohort may also have been a contributing factor to these findings.

This study also found that patients with vitiligo receive more outpatient services and thus incur higher costs versus patients without vitiligo. Increased outpatient visits may result in time away from work and thus may reduce work productivity and increase the indirect cost burden for patients.^24^ These results are consistent with a recent US retrospective database claims study that found all-cause costs and HCRU were higher for patients with vitiligo versus non-vitiligo control patients; however, in contrast to our study, the US study found mental health HCRU was also higher among patients with vitiligo.^25^

Higher total service costs were also observed for patients with vitiligo 1 year after their index date. It has been previously shown that vitiligo-associated costs vary depending on treatment.^26^ The lack of a consistent treatment approach was indicated by 43 different treatment sequences. More than a third of patients had ≥4 lines of treatment, indicating a lack of effective options. Treatment adherence and persistence were low but may be underestimated because most treatments were topicals, and 1 prescription (ie, tube) could likely be used for an extended period.

This study was limited in part by its reliance on *ICD-9/10* codes for medical service. First, this approach assumes the correct coding of diseases in the *ICD-9/10* code by physicians. Second, *ICD-9* classification has no unique diagnostic code for vitiligo and instead is grouped together with other skin diseases. Nevertheless, we used a validated algorithm to distinguish vitiligo cases. No patients were identified using the *ICD-10* code L80.x for vitiligo since this updated coding system has not yet been implemented in Québec for medical claim reimbursement. Patients were instead identified according to treatment, which may have underestimated the number of patients with vitiligo, as some patients with vitiligo may not seek medical care because of perceived lack of efficacious therapies. Furthermore, as data do not capture the specific indication for prescribed treatment, vitiligo-related treatments may have been prescribed for other medical conditions. In addition, variability in the determination of medication supply for topical therapies (estimated by pharmacists) may have affected the interpretation of treatment patterns with these agents. Also, this analysis does not capture data for BSA, involvement of special sites, or Fitzpatrick skin type, which may exacerbate QoL burden.^7,8^ Last, only patients with a public health plan are represented in this study, which accounts for approximately 40% of the Québec population, and may disproportionally capture data from specific population subsets (eg, patients aged ≥65 years). Future studies would benefit from the use of data sources that include larger and more representative sections of the population.

In summary, the results of this study demonstrate a part of the burden and HCRU among patients with vitiligo in Québec, Canada, and indicate a need for a multidisciplinary approach to vitiligo management. Specific vitiligo treatment guidelines are needed for Canada, as current treatments may be heterogeneous depending on current literature, patient preferences, and disease severity.^27^

## Supplemental Material

sj-docx-1-cms-10.1177_12034754241304683 – Supplemental material for Burden of Vitiligo in Canada: Retrospective Analysis of a Canadian Public Claims Database Supplemental material, sj-docx-1-cms-10.1177_12034754241304683 for Burden of Vitiligo in Canada: Retrospective Analysis of a Canadian Public Claims Database by Julien Ringuet, Grace K. Wong, Véronique Baribeau, Sunil Kalia, Josée Brisebois and Jean Lachaine in Journal of Cutaneous Medicine and Surgery
