# Supplementary material for: Burden of Vitiligo in Canada: Retrospective Analysis of a Canadian Public Claims Database
Source: J Cutan Med Surg. 2025 Jan 22;29(3):234–42. doi: 10.1177/12034754241304683 (PMC12171071; doi:10.1177/12034754241304683)
Supplement: sj-docx-1-cms-10.1177_12034754241304683 – Supplemental material for Burden of Vitiligo in Canada: Retrospective Analysis of a Canadian Public Claims Database [file sj-docx-1-cms-10.1177_12034754241304683.docx]

# Supplementary Appendix

**Burden of Vitiligo in Canada: Retrospective Analysis of a Canadian Public Claims Database**

Julien Ringuet, MD, MSc, Grace K. Wong, PhD, Véronique Baribeau, MSc, Sunil Kalia, MD, Josée Brisebois, PhD, Jean Lachaine, PhD

Figure S1. Patient Selection. *ICD*, *International Classification of Diseases*. ^†^ Date of the first other skin disease. ^‡^ Date of the first medical service.


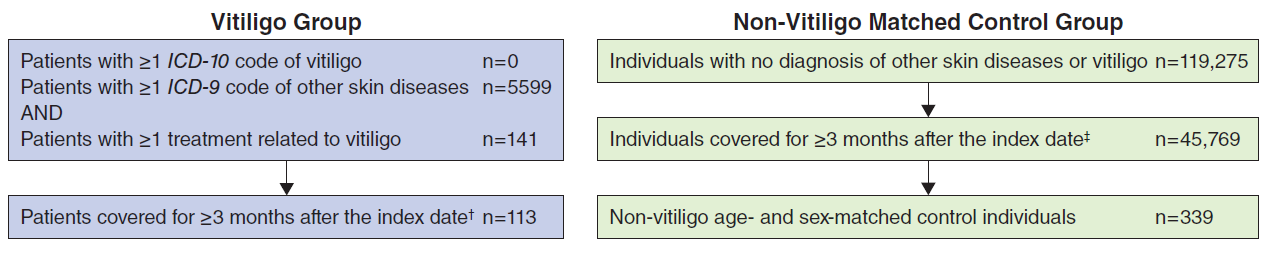


**Figure S2. Seasonality of Prescriptions of Treatment for Patients With Vitiligo.** TCS, topical corticosteroids.


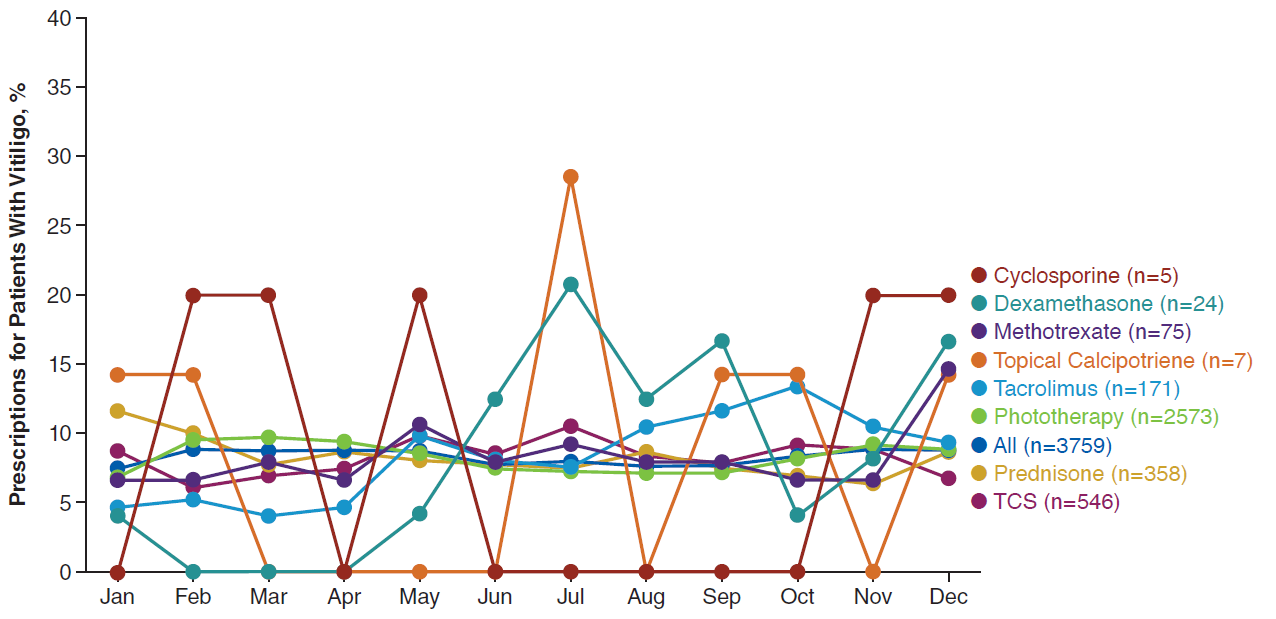


Table S1. Demographic Characteristics of the Vitiligo and the Non-Vitiligo Matched Control Group

| **Characteristic^a^** | Vitiligo  (n=113) | Control  (n=339) | *P* value |
| --- | --- | --- | --- |
| Female, n (%) | 77 (68.1) | 231 (68.1) | 1.00 |
| Age at index date, mean (SD), y | 50.0 (24.9) | 50.4 (25.3) | 0.89 |
| Age category, n (%), y |  |  |  |
| <20 | 19 (16.8) | 57 (16.8) | 1.00 |
| 20–34 | 13 (11.5) | 39 (11.5) | 1.00 |
| 35–49 | 18 (15.9) | 54 (15.9) | 1.00 |
| 50–64 | 17 (15.0) | 51 (15.0) | 1.00 |
| ≥65 | 46 (40.7) | 138 (40.7) | 1.00 |
| Type of drug plan in 2018, n (%) |  |  |  |
| ≥65+ y | 48 (42.5) | 168 (49.6) | 0.19 |
| Adherent^b^ | 57 (50.4) | 139 (41.0) | 0.08 |
| Social welfare | 8 (7.1) | 32 (9.4) | 0.44 |
| Geographic location in 2018,  n (%) |  |  |  |
| Montréal-Centre | 43 (38.1) | 74 (21.8) | **<0.01** |
| Monteregie | 10 (8.8) | 57 (16.8) | **0.04** |
| Québec City | 19 (16.8) | 20 (5.9) | **<0.01** |
| Mauricie and Central Québec | 2 (1.8) | 30 (8.8) | **0.01** |
| Chaudiere-Appalaches | 4 (3.5) | 24 (7.1) | 0.26 |
| Laurentians | 4 (3.5) | 20 (5.9) | 0.33 |
| Laval | 5 (4.4) | 19 (5.6) | 0.63 |
| Lanaudiere | 5 (4.4) | 16 (4.7) | 0.90 |
| Eastern Townships | 4 (3.5) | 16 (4.7) | 0.60 |
| Saguenay –Lac-Saint-Jean | 3 (2.7) | 15 (4.4) | 0.58 |
| Gaspesia and Magdalen Islands | 6 (5.3) | 8 (2.4) | 0.13 |
| Lower St. Lawrence | 1 (0.9) | 11 (3.2) | 0.31 |
| Ottawa Valley | 3 (2.7) | 9 (2.7) | 1.00 |
| North Shore | 3 (2.7) | 7 (2.1) | 0.72 |
| Abitibi-Temiscamingue | 1 (0.9) | 8 (2.4) | 0.46 |
| Northern Québec | 0 | 1 (0.3) | 1.00 |
| Cree territory of James Bay | 0 | 0 | – |
| Nunavik | 0 | 0 | – |
| Not available | 0 | 4 (1.2) | 0.58 |
| Follow-up, mean (SD), y | 4.2 (2.6) | 7.2 (3.6) | **<0.01** |

^a^ Patient characteristics available only in 2018 database. Age at index date was calculated from age in 2018.

^b^ Individuals not admissible to a private drug plan at their workplace.

Table S2. Sequences of Vitiligo Treatments in Patients With Vitiligo

| **Sequences^a^** | n (%) |
| --- | --- |
| No sequence^b^ | 31 (27.4) |
| Phototherapy | 12 (10.6) |
| TCS | 10 (8.8) |
| Tacrolimus | 8 (7.1) |
| TCS 🡪 TCS | 6 (5.3) |
| TCS 🡪 Phototherapy | 4 (3.5) |
| TCS 🡪 TCS 🡪 Tacrolimus | 3 (2.7) |
| Prednisone | 2 (1.8) |
| Tacrolimus 🡪 Tacrolimus | 2 (1.8) |
| TCS 🡪 Tacrolimus | 2 (1.8) |
| Calcipotriene + Tacrolimus + TCS 🡪 Calcipotriene | 1 (0.9) |
| Phototherapy 🡪 Methotrexate | 1 (0.9) |
| Phototherapy 🡪 Phototherapy + TCS | 1 (0.9) |
| Phototherapy 🡪 TCS | 1 (0.9) |
| Phototherapy 🡪 Phototherapy + TCS 🡪 TCS | 1 (0.9) |
| Phototherapy 🡪 Phototherapy + TCS 🡪 TCS 🡪 Phototherapy🡪 Phototherapy + Tacrolimus | 1 (0.9) |
| Phototherapy 🡪 TCS 🡪 Phototherapy 🡪 Phototherapy + TCS 🡪 Phototherapy 🡪 Phototherapy 🡪 Phototherapy + TCS 🡪 Phototherapy 🡪 TCS | 1 (0.9) |
| Phototherapy 🡪 Phototherapy + Tacrolimus 🡪 TCS 🡪 Tacrolimus 🡪 Tacrolimus + TCS 🡪 TCS 🡪 TCS 🡪 Tacrolimus 🡪 Tacrolimus + TCS 🡪 Tacrolimus | 1 (0.9) |
| Pimecrolimus 🡪 Pimecrolimus 🡪 Pimecrolimus 🡪 TCS | 1 (0.9) |
| Prednisone 🡪 TCS | 1 (0.9) |
| Prednisone + TCS 🡪 Prednisone 🡪 TCS 🡪 Tacrolimus 🡪 TCS | 1 (0.9) |
| Prednisone 🡪 Prednisone + Methotrexate 🡪 Dexamethasone 🡪 Prednisone 🡪 Prednisone 🡪 TCS | 1 (0.9) |
| Tacrolimus + TCS 🡪 Tacrolimus | 1 (0.9) |
| Tacrolimus 🡪 TCS 🡪 TCS | 1 (0.9) |
| Tacrolimus + TCS 🡪 Tacrolimus 🡪 TCS | 1 (0.9) |
| Tacrolimus + Cyclosporine 🡪 Tacrolimus 🡪 Tacrolimus + TCS + Cyclosporine 🡪 Tacrolimus | 1 (0.9) |
| TCS 🡪 Methotrexate 🡪 Phototherapy | 1 (0.9) |
| TCS 🡪 Phototherapy 🡪 TCS | 1 (0.9) |
| TCS 🡪 TCS 🡪 Methotrexate | 1 (0.9) |
| TCS 🡪 TCS 🡪 Phototherapy 🡪 Phototherapy + Prednisone | 1 (0.9) |
| TCS 🡪 TCS + Prednisone 🡪 Prednisone 🡪 Prednisone | 1 (0.9) |
| TCS 🡪 TCS 🡪 TCS 🡪 TCS | 1 (0.9) |
| TCS 🡪 Prednisone 🡪 TCS 🡪 Prednisone 🡪 Tacrolimus | 1 (0.9) |
| TCS 🡪 TCS + Prednisone 🡪 Phototherapy 🡪 Phototherapy + Prednisone 🡪 Prednisone | 1 (0.9) |
| TCS 🡪 TCS 🡪 TCS 🡪 TCS 🡪 Phototherapy | 1 (0.9) |
| TCS 🡪 TCS 🡪 TCS 🡪 TCS 🡪 TCS | 1 (0.9) |
| TCS 🡪 TCS 🡪 Phototherapy 🡪 TCS 🡪 TCS 🡪 TCS | 1 (0.9) |
| TCS 🡪 TCS + Tacrolimus 🡪 Tacrolimus 🡪 Tacrolimus + TCS 🡪 Phototherapy 🡪 Tacrolimus | 1 (0.9) |
| TCS 🡪 TCS 🡪 Phototherapy 🡪 TCS 🡪 Phototherapy 🡪 Phototherapy 🡪 Phototherapy + TCS 🡪 TCS | 1 (0.9) |
| TCS 🡪 TCS 🡪 TCS 🡪 Phototherapy 🡪 TCS 🡪 TCS 🡪 TCS 🡪 TCS | 1 (0.9) |
| TCS 🡪 Phototherapy 🡪 Phototherapy + Prednisone 🡪 Prednisone 🡪 Prednisone 🡪 Prednisone 🡪 Prednisone + Calcipotriene 🡪 Phototherapy 🡪 TCS | 1 (0.9) |
| TCS + Tacrolimus 🡪 TCS 🡪 TCS 🡪 TCS + Tacrolimus 🡪 Tacrolimus 🡪 TCS 🡪 TCS 🡪 Tacrolimus 🡪 Tacrolimus | 1 (0.9) |
| TCS🡪 TCS 🡪 Prednisone 🡪 Prednisone + TCS 🡪 TCS 🡪 TCS + Prednisone 🡪 Prednisone 🡪 Phototherapy 🡪 TCS 🡪 TCS 🡪 Phototherapy | 1 (0.9) |

TCS, topical corticosteroids.

^a^Treatment episodes ≥30 days were considered as part of a sequence. Treatments listed twice or more in the same sequence were restarted after a period of discontinuation (gap of dispensation for ≥90 days).

^b^ No treatment lasted ≥30 days.

Table S3. Adherence and Persistence for Vitiligo-Related Treatments

| Adherence and Persistence | TCS | Tacrolimus | Topical Calcipotriene | Prednisone | Dexamethasone | Methotrexate |
| --- | --- | --- | --- | --- | --- | --- |
| 3 months |  |  |  |  |  |  |
| Adherence, n | 25 | 7 | 0 | 5 | 1 | 4 |
| Mean (SD) MPR, % | 57.9 (29.5) | 37.1 (25.4) | – | 50.2 (34.8) | 100 | 98.3 (3.3) |
| Adherent,^a^ n (%) | 7 (28.0) | 1 (14.3) | – | 1 (20.0) | 1 (100) | 4 (100) |
| Persistence, n | 77 | 46 | 5 | 27 | 3 | 5 |
| Discontinuation rate, % (95% CI) | 65.5 (54.9–76.2) | 80.9 (69.0–92.9) | 80.0  (44.9–100) | 78.8  (63.0–94.6) | 66.7  (13.3–100) | 0  (0–0) |
| 6 months |  |  |  |  |  |  |
| Adherence, n | 10 | 4 | 0 | 4 | 1 | 3 |
| Mean (SD) MPR, % | 65.8 (30.4) | 47.8 (19.4) | – | 55.1 (30.3) | 100 | 100 |
| Adherent,^a^ n (%) | 1 (25.0) | – | – | 0 | – | 1 (100) |
| Persistence, n | 77 | 46 | 5 | 27 | 3 | 5 |
| Discontinuation rate, % (95% CI) | 86.2  (78.3–94.1) | 86.4  (75.7–97.1) | 80.0  (44.9–100) | 83.1  (68.4–97.7) | 66.7  (13.3–100) | 25.0  (0–67.4) |
| 12 months |  |  |  |  |  |  |
| Adherence, n | 4 | 0 | 0 | 1 | 0 | 1 |
| Mean (SD) MPR, % | 74.7 (18.0) | – | – | 67.4 | – | 80.5 |
| Adherent,^a^ n (%) | 1 (25.0) | – | – | 0 | – | 1 (100) |
| Persistence, n | 77 | 46 | 5 | 27 | 3 | 5 |
| Discontinuation rate, % (95% CI) | 93.8  (88.1–99.5) | 100  (100–100) | 80.0  (44.9–100) | 95.8  (87.7–100) | 100  (100–100) | 50.0  (1.0–99.0) |
| Mean (SD) time to discontinuation, d | 112 (25) | 68 (15) | 32 (10) | 76 (27) | 85 (83) | 725 (285) |

MPR, medication possession ratio; TCS topical corticosteroids.

^a^ Defined as MPR ≥80%.

Table S4. Adherence and Persistence for Vitiligo-Related Treatments: 180-Day Discontinuation Gap for Sensitivity Analysis

| Adherence and Persistence | TCS | Tacrolimus | Topical Calcipotriene |
| --- | --- | --- | --- |
| 3 months |  |  |  |
| Adherence, n | 33 | 9 | 1 |
| Mean (SD) MPR, % | 50.1 (29.4) | 31.6 (24.7) | 22.2 (–) |
| Adherent,^a^ n (%) | 7 (21.2) | 1 (11.1) | 0 |
| Persistence, n | 77 | 46 | 5 |
| Discontinuation rate, % (95% CI) | 55.0 (43.8–66.2) | 76.3 (63.4–89.1) | 60.0 (17.1–100) |
| 6 months |  |  |  |
| Adherence, n | 20 | 6 | 0 |
| Mean (SD) MPR, % | 45.6 (31.0) | 42.5 (18.2) | – |
| Adherent,^a^ n (%) | 5 (25.0) | 0 | – |
| Persistence, n | 77 | 46 | 5 |
| Discontinuation rate, % (95% CI) | 72.7 (61.5–83.9) | 81.5  (69.7–93.4) | 60.0  (17.1–100) |
| 12 months |  |  |  |
| Adherence, n | 10 | 3 | 0 |
| Mean (SD) MPR, % | 49.3 (29.9) | 42.4 (13.0) | – |
| Adherent,^a^ n (%) | 1 (10.0) | 0 | – |
| Persistence, n | 77 | 46 | 5 |
| Discontinuation rate, % (95% CI) | 84.2  (75.7–92.6) | 88.9  (78.3–99.6) | 60.0  (17.1–100) |
| Mean (SD) time to discontinuation, d | 186 (36) | 187 (74) | 81 (38) |

MPR, medication possession ratio; TCS topical corticosteroids.

^a^ Defined as MPR ≥80%.

Table S5. Healthcare Resource Utilization and Costs for Vitiligo-Related Resources During the Follow-Up in Patients With Vitiligo

| **Vitiligo-Related HCRU** | Patients With Vitiligo  (n=113) |
| --- | --- |
| Annualized inpatient visits |  |
| Patients with ≥1 visit per year, n (%) | 2 (1.8) |
| Cost, mean (SD), CAN$ | 47 (437) |
| Annualized ED visits |  |
| Patients with ≥1 visit per year, n (%) | 3 (2.7) |
| Cost, mean (SD), CAN$ | 2 (13) |
| Annualized outpatient visits |  |
| Patients with ≥1 visit per year, n (%) | 109 (96.5) |
| Cost, mean (SD), CAN$ | 123 (274) |
| Annualized other visits |  |
| Patients with ≥1 visit per year, n (%) | 3 (2.7) |
| Cost, mean (SD), CAN$ | 0.3 (3) |
| Annualized total costs, mean (SD), CAN$ |  |
| Total services cost | 172 (523) |
| Total medications cost | 90 (189) |
| Total health care services | 263 (568) |

ED, emergency department.
